# Recurrent Multi-territory Thromboembolism As the Initial Presentation of Pancreatic Adenocarcinoma and Suspected Nonbacterial Thrombotic Endocarditis

**DOI:** 10.7759/cureus.119560

**Published:** 2026-11-12

**Authors:** Rasha Alsammak, Peter Ferry

**Affiliations:** 1 Department of Geriatric Medicine, Karin Grech Rehabilitation Hospital, Pietà, MLT

**Keywords:** embolic stroke, non-bacterial thrombotic endocarditis (nbte), pancreatic adenocarcinoma, recurrent thromboembolism, trousseau syndrome

## Abstract

Nonbacterial thrombotic endocarditis (NBTE), also known as Marantic endocarditis, is an uncommon complication of malignancy that may cause systemic embolization. We report a 74-year-old woman who developed a right gastrocnemius deep vein thrombosis followed by recurrent right middle cerebral artery occlusions requiring two mechanical thrombectomies. Her subsequent course was complicated by further deep vein thrombosis, pulmonary embolism, and acute limb ischemia. Repeat echocardiography demonstrated mitral valve vegetations, initially raising concern for infective endocarditis. However, repeated blood cultures and cultures of retrieved thrombotic material were negative. Imaging revealed widespread metastatic disease, and biopsy of a left supraclavicular lymph node confirmed metastatic pancreatic adenocarcinoma. The combination of recurrent arterial and venous thrombosis, advanced malignancy, and negative microbiological investigations favored a clinical diagnosis of NBTE. This case illustrates how extensive thromboembolic disease may precede the diagnosis of pancreatic cancer and highlights the diagnostic challenge posed by valvular vegetations in this setting.

## Introduction

Malignancy-associated thrombosis, commonly described as Trousseau syndrome, is particularly associated with mucin-producing adenocarcinomas, including pancreatic cancer [1,2]. Tumor-mediated activation of coagulation, platelets, and inflammatory pathways creates a hypercoagulable state that may cause both venous and arterial thromboembolic events [2]. These manifestations can precede cancer diagnosis and provide an important clue to an underlying malignancy [1,2].

Nonbacterial thrombotic endocarditis (NBTE) is a related complication characterized by sterile platelet-fibrin vegetations on cardiac valves, which may embolize to the brain and other systemic vascular territories [3,4]. Unlike infective endocarditis (IE), NBTE occurs without microbial infection of the valve. However, its clinical and echocardiographic features can overlap with those of IE, making diagnosis challenging [3,4]. We describe a patient whose recurrent venous thrombosis and systemic embolic events led to the diagnosis of metastatic pancreatic adenocarcinoma and clinically suspected NBTE. The educational value of this case lies in the evolving combination of venous and arterial events before recognition of the underlying malignancy, the diagnostic uncertainty surrounding valvular vegetations, and the challenge of balancing anticoagulation against intracranial bleeding risk. It highlights the importance of considering occult malignancy and NBTE when recurrent venous thrombosis is accompanied by systemic arterial embolization.

## Case presentation

A 74-year-old woman with a history of hypertension, who was living independently at baseline, initially presented to the emergency department with right calf discomfort. Duplex ultrasonography confirmed a deep vein thrombosis (DVT) involving the right medial gastrocnemius vein. She was started on therapeutic rivaroxaban 15 mg twice daily.

Three weeks later, she presented with acute left-sided hemiplegia, facial deviation, and left-sided neglect. Imaging confirmed a right middle cerebral artery (MCA) occlusion and a small-volume subarachnoid hemorrhage (SAH) (Figure 1). She underwent successful mechanical thrombectomy. In view of the concomitant intracranial hemorrhage and the need to determine an appropriate antithrombotic strategy, repeat venous Doppler ultrasonography was performed and demonstrated no extension of the known DVT. Following discussion with the hematology team, she started aspirin 75 mg once daily and prophylactic-dose enoxaparin, with a planned minimum duration of six weeks, before being discharged to the rehabilitation facility.

**Figure 1:**
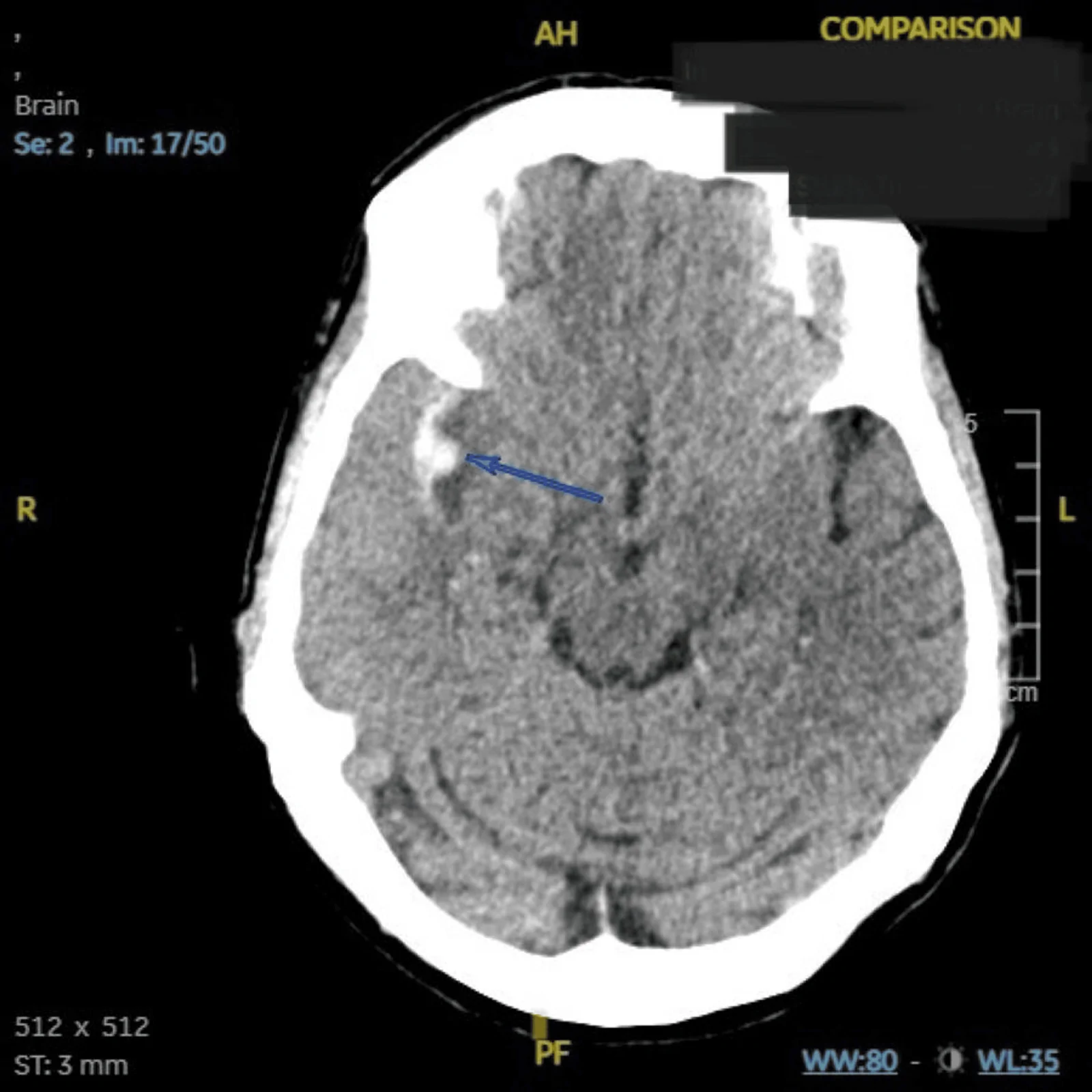
Brain CT showing a small-volume SAH (arrow). CT, computed tomography; SAH, subarachnoid hemorrhage

Four weeks into rehabilitation, she developed recurrent left-sided hemiplegia and neglect; CT stroke imaging demonstrated a second right MCA occlusion (Figure 2). A repeat mechanical thrombectomy was successfully performed, and postprocedural imaging revealed a 5-mm intracerebral hemorrhage.

**Figure 2:**
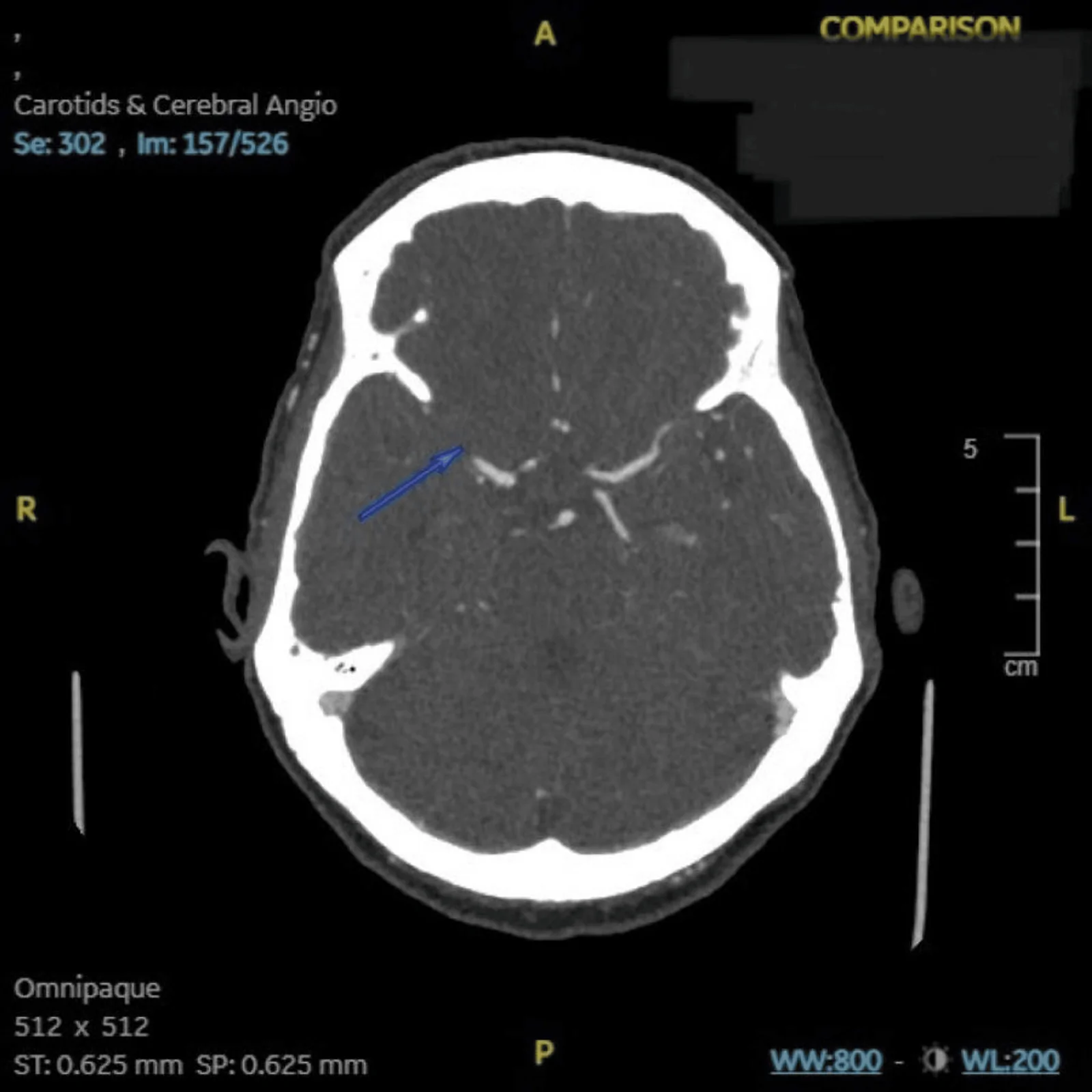
CT imaging of the patient showing right MCA occlusion (arrow). CT, computed tomography; MCA, middle cerebral artery

Twenty-four-hour cardiac monitoring showed no evidence of atrial arrhythmia. Carotid Arteries Duplex Scan showed no significant stenosis. Transthoracic echocardiography (TTE) demonstrated preserved left ventricular systolic function (ejection fraction 75%), degenerative aortic valve disease with mild aortic stenosis, moderate mitral regurgitation, mild tricuspid regurgitation, a dilated left atrium, and an aneurysmal interatrial septum without a definite shunt on color Doppler imaging. She was discharged back to rehabilitation on clopidogrel 75 mg once daily and prophylactic-dose enoxaparin, with an outpatient bubble-contrast echocardiogram planned to exclude an intracardiac shunt.

One week later, she developed left calf discomfort. Duplex ultrasonography confirmed a new left distal femoral (Figure 3) and popliteal vein thrombosis with associated superficial thrombophlebitis (Figure 4). On hematology advice, warfarin therapy was initiated with therapeutic-dose enoxaparin bridging, targeting an INR of 2-3, and clopidogrel was discontinued.

**Figure 3:**
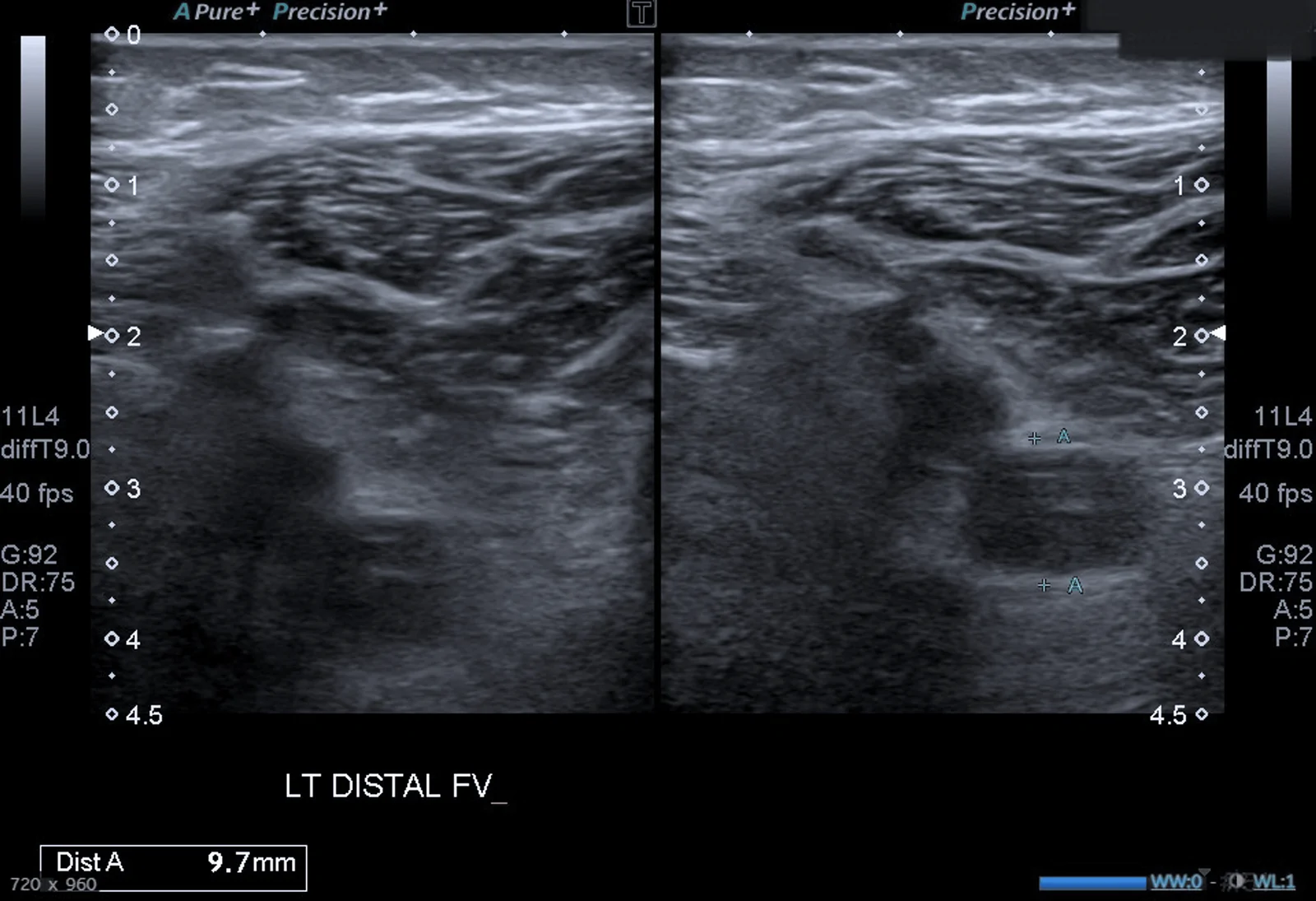
Duplex ultrasound of the patient showing left distal femoral vein thrombosis (blue indicators). FV, femoral vein

**Figure 4:**
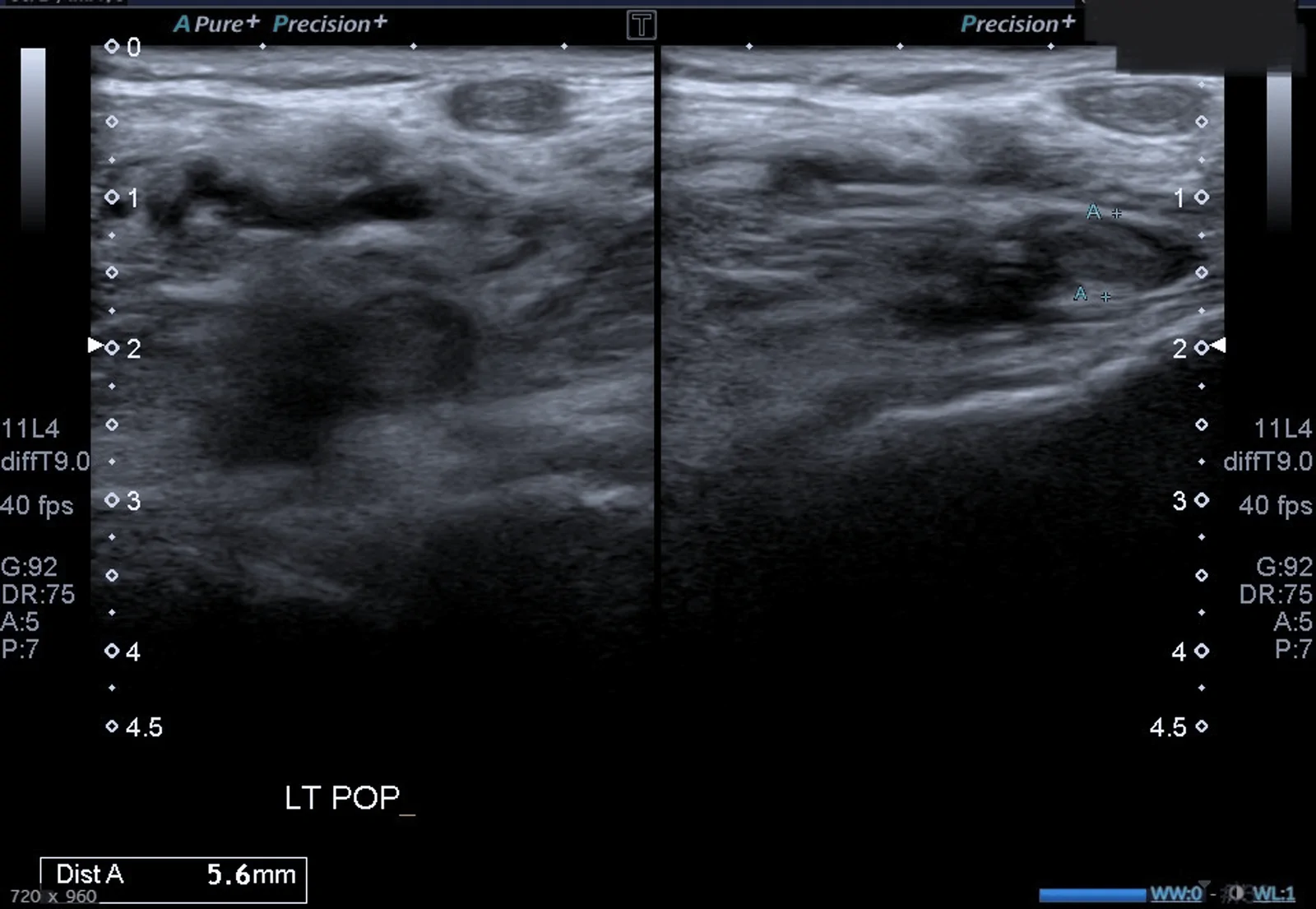
Duplex ultrasound of the patient showing popliteal vein thrombosis (blue indicators).

During this admission, she developed persistent low-grade fever (up to 38°C) with a moderately elevated C-reactive protein (CRP). Septic work-up, including chest X-ray, respiratory pathogen panel, urinalysis, urine culture, and blood cultures, was unrevealing.

Serum virology tests and immunological investigations, including anticardiolipin antibodies, anti-β2-glycoprotein I antibodies, ANA, and rheumatoid factor, were negative.

Empirical antibiotics were initiated with co-amoxiclav and later escalated to piperacillin-tazobactam due to ongoing fevers.

While undergoing further investigations, a left supraclavicular lymph node was noted on clinical examination; an urgent CT of the neck, chest, abdomen, and pelvis was planned. On the same day, she was assessed by a cardiologist, and repeat echocardiography revealed mitral valve vegetations (Video 1), including a large sessile vegetation on the atrial aspect of the posterior mitral leaflet and a smaller vegetation on the anterior leaflet (Figure 5), with associated moderate mitral regurgitation. IE was therefore considered a significant possibility, and a transesophageal echocardiogram (TEE) was planned; a bubble-contrast study was deemed unnecessary.

**Video 1:** TTE of the patient showing vegetations on the anterior and posterior leaflets of the mitral valve. TTE, transthoracic echocardiogram

**Figure 5:**
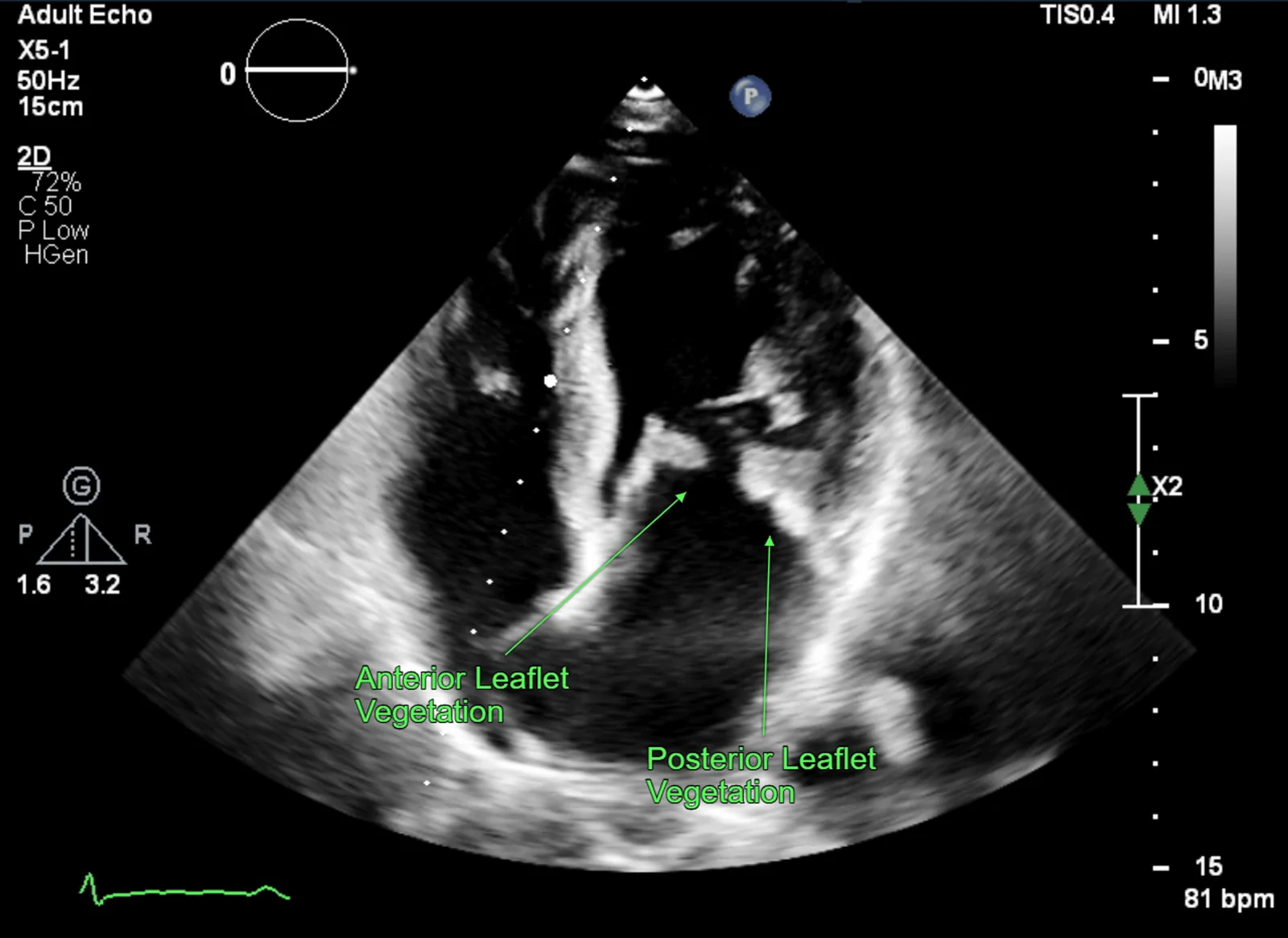
TTE view indicating the vegetations in the anterior and posterior leaflets (arrows) of the mitral valve. TTE, transthoracic echocardiogram

The concurrent cross-sectional imaging demonstrated widespread metastatic disease involving the liver (Figure 6), with a possible pancreatic primary malignancy (Figure 7), in addition to a subsegmental pulmonary embolism, femoral vein thrombosis, and suspected splenic and renal infarctions. These findings raised the possibility of an underlying malignancy-associated hypercoagulable state and NBTE as an alternative explanation for the valvular vegetations and recurrent systemic embolization.

**Figure 6:**
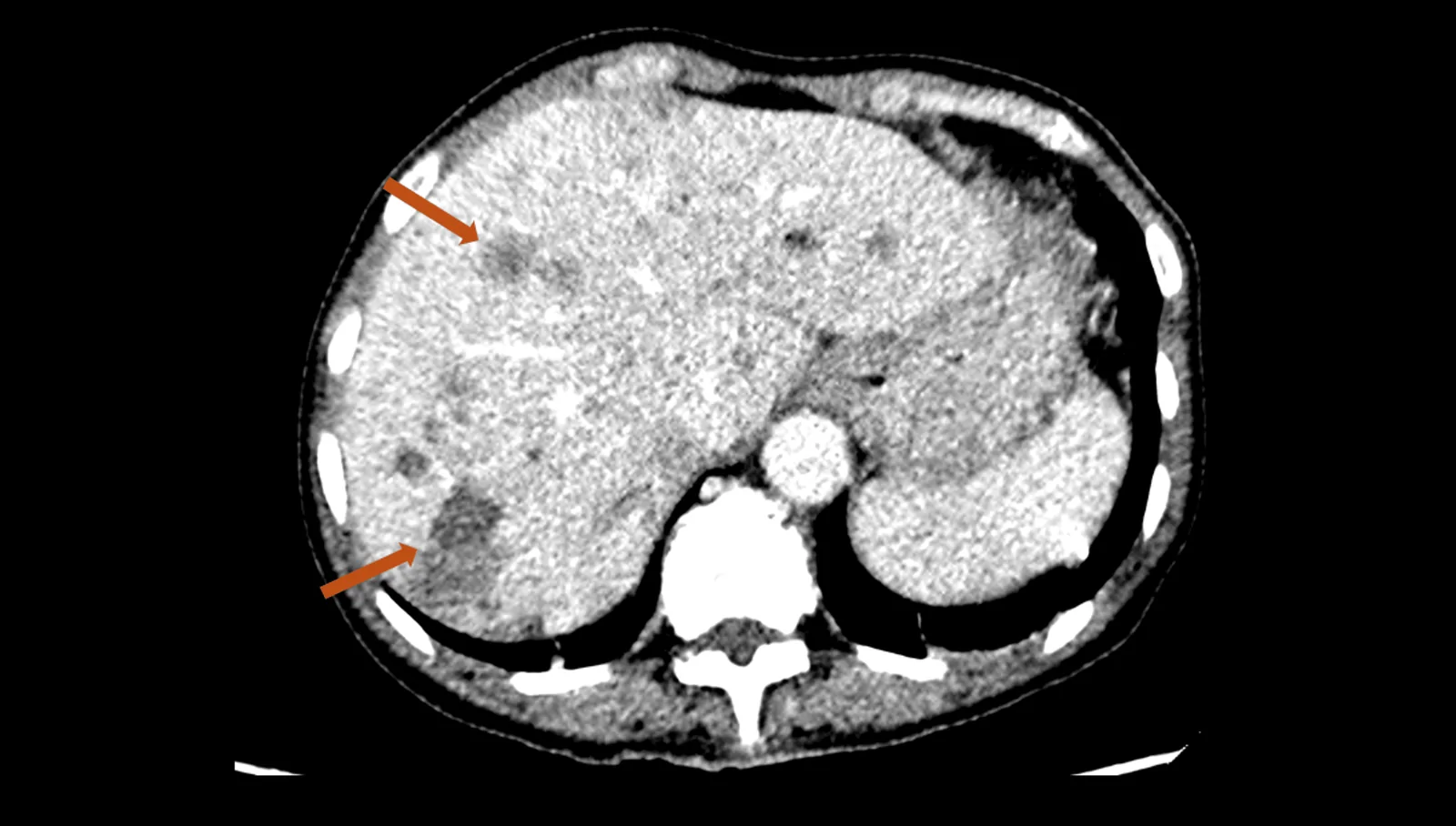
Contrast-enhanced CT of the patient's abdomen demonstrating multiple bilobar hypodense lesions of variable sizes within the liver, in keeping with hepatic metastases (arrows). CT, computed tomography

**Figure 7:**
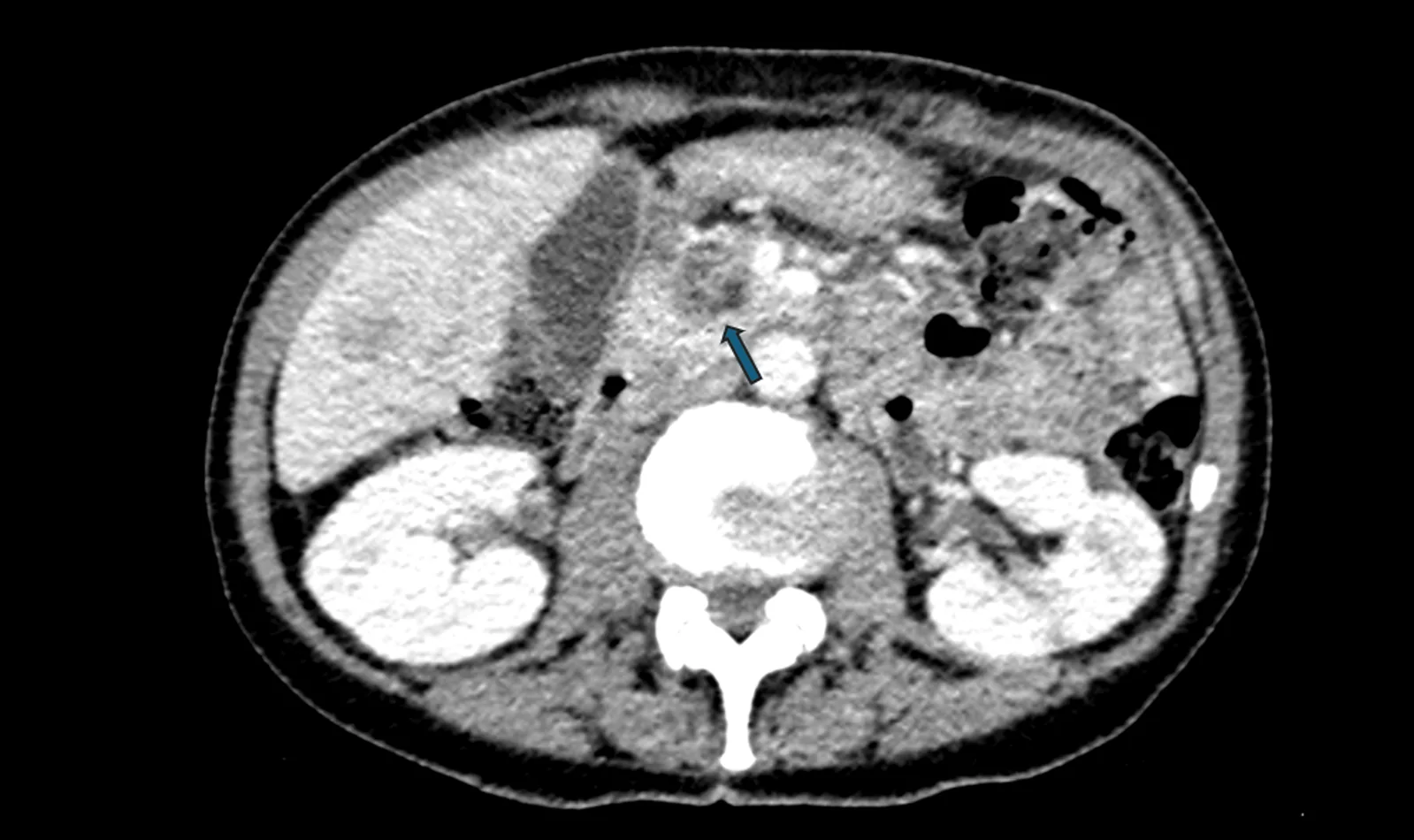
Contrast-enhanced CT of the patient's abdomen revealed a hypodense lesion in the pancreatic head measuring 1.9 cm (arrow). CT, computed tomography

Another three sets of blood cultures were sent. Vancomycin was commenced in addition to piperacillin-tazobactam after discussion with the infectious diseases team. Given the newly identified malignancy, the possibility that the vegetations represented a manifestation of the patient’s prothrombotic state was also considered.

The anticoagulation strategy was subsequently revisited with hematology in view of the malignancy diagnosis, and warfarin was switched to therapeutic-dose enoxaparin.

In total, 13 sets of blood cultures were obtained. Cultures from a single day yielded growth in two of three bottles, with Staphylococcus hominis, Staphylococcus capitis, and Staphylococcus epidermidis isolated. These organisms were considered more likely to represent skin contamination in the overall clinical context. Tests for Coxiella, Brucella, and fungal infection were all negative.

The infectious diseases team considered NBTE to be the most likely diagnosis. However, as IE could not be definitively excluded, antimicrobial therapy was continued.

Subsequently, a biopsy of the left supraclavicular lymph node confirmed metastatic moderately to poorly differentiated pancreatic adenocarcinoma. On the same day as the biopsy, the patient developed acute right limb ischemia requiring successful thrombectomy of the right common femoral artery; microbiological culture of the retrieved thrombus was negative for bacterial growth.

Taken together, the recurrent venous and arterial thromboembolic events, widespread metastatic pancreatic adenocarcinoma, and presence of mitral valve vegetations in the setting of persistently negative microbiological investigations were considered most consistent with malignancy-associated hypercoagulability complicated by NBTE.

The planned TEE was not performed due to the patient's rapid clinical deterioration and transition to palliative care.

Following multidisciplinary discussion with oncology and the treating teams, the patient was considered unsuitable for definitive oncological treatment because of her advanced disease and deteriorating clinical condition. The decision to transition to palliative care was discussed with the patient and her relatives. She was subsequently transferred to a palliative care unit for symptom control and comfort-focused management. She died approximately three months after her initial presentation.

The timeline of the clinical events and antithrombotic management is summarized chronologically in Table 1.

**Table 1:** Timeline of events. BID, twice daily; CT, computed tomography; DVT, deep vein thrombosis; INR, international normalized ratio; MCA, middle cerebral artery; SAH, subarachnoid hemorrhage

Time from initial presentation	Clinical events	Antithrombotic therapy
Day 0	Right DVT	Rivaroxaban started 15 mg BID
Week 3	Right MCA stroke, SAH	Aspirin 75 mg + prophylactic enoxaparin
Week 7	Recurrent right MCA stroke	Clopidogrel 75 mg + prophylactic enoxaparin
Week 8	Left DVT	Warfarin with therapeutic enoxaparin bridging (INR aim of 2-3 was achieved)
Week 9	CT highly suggestive of malignancy	Enoxaparin therapeutic dose (received for 7 days before the next event)
Week 10	Acute limb ischemia	Heparin infusion during perioperative period of thrombectomy
Week 11	Histology confirmed metastatic pancreatic adenocarcinoma, clinical deterioration and transition to palliative care	Enoxaparin therapeutic dose
Week 12	Death	

## Discussion

We describe a patient who presented with recurrent venous and arterial thromboembolic events preceding the diagnosis of metastatic pancreatic adenocarcinoma and in whom mitral valve vegetations were subsequently identified and considered most consistent with NBTE. The patient developed sequential deep vein thromboses, pulmonary embolism, recurrent right middle cerebral artery occlusions, suspected visceral infarctions, and acute limb ischemia, with recurrent events occurring despite anticoagulation with different agents.

This pattern is characteristic of malignancy-associated thrombosis and should prompt consideration of occult malignancy and NBTE in patients with recurrent thromboembolic events involving multiple vascular territories, particularly when conventional cardioembolic sources are not identified [1,4].

Trousseau syndrome encompasses a spectrum of malignancy-associated thrombotic manifestations and is classically associated with mucin-producing adenocarcinomas, particularly pancreatic adenocarcinoma [1]. Venous thromboembolism is a well-recognized complication of malignancy, while arterial thromboembolic events, including ischemic stroke, also contribute to morbidity and mortality [5]. In some patients, thrombotic manifestations precede the diagnosis of cancer by weeks or months, making unexplained or recurrent thrombosis a potential clue to occult malignancy [6,7].

Trousseau syndrome and NBTE are closely related manifestations of cancer-associated hypercoagulability. Trousseau syndrome encompasses systemic thrombotic complications, whereas NBTE refers specifically to sterile platelet-fibrin vegetations on cardiac valves that may result in systemic embolization [1,4]. In our patient, recurrent DVTs and pulmonary embolism reflected systemic hypercoagulability, while mitral valve vegetations provided a plausible source of recurrent cerebral emboli and potentially the acute limb ischemia. Their coexistence was consistent with the profound prothrombotic state associated with advanced pancreatic cancer.

NBTE is an uncommon but recognized complication of malignancy [4]. In a contemporary Cleveland Clinic cohort of 42 patients, malignancy was identified in 40.5%, systemic lupus erythematosus in 33.3%, and antiphospholipid syndrome in 35.7%. Stroke was the most common presentation, occurring in 59.5%, and the mitral valve was most frequently involved, affecting 61.9% [8]. Pancreatic cancer was among the commonly reported malignancies associated with NBTE in the 2023 ESC guideline [9]. These findings are consistent with the present case, in which recurrent embolic stroke was the dominant clinical presentation and mitral valve vegetations were subsequently identified.

Diagnostic challenge of NBTE versus IE

Distinguishing NBTE from IE represented the principal diagnostic challenge. Echocardiographic evidence of mitral valve vegetations, together with recurrent arterial embolic phenomena, raised substantial concern for IE. This combination was compatible with possible IE under the 2023 Duke-ISCVID criteria [10].

However, several findings reduced the likelihood of IE, including repeatedly negative blood cultures. Blood cultures remained repeatedly negative apart from coagulase-negative staphylococci isolated from two of three bottles obtained on a single day, which were considered likely contaminants. Cultures obtained from retrieved embolic material were also negative, and serological investigations for recognized causes of blood culture-negative endocarditis, including Coxiella burnetii and Brucella spp., were unrevealing. Although these results did not independently exclude IE, they reduced its likelihood and supported consideration of a noninfective cause in the clinical context [4,9].

Conversely, several features favored NBTE. The patient had newly diagnosed metastatic pancreatic adenocarcinoma, a recognized predisposing condition [4,9]. Mitral valve vegetations were identified without reported evidence of valvular destruction or leaflet perforation. These findings supported NBTE, although echocardiographic appearances alone cannot reliably distinguish sterile from infective vegetations [4]. Negative immunological investigations also provided no evidence for an associated autoimmune cause.

Initial TTE did not demonstrate vegetations, which became apparent on repeat imaging approximately 12 days later. This illustrates the need for repeat imaging and consideration of TEE when clinical suspicion persists despite a negative TTE [4,8].

In a comparative study of patients with systemic lupus erythematosus, TTE had a sensitivity of 63%, specificity of 58%, and negative predictive value of 40% for Libman-Sacks endocarditis when TEE was used as the reference standard; sensitivity for vegetations alone was only 11% [11]. These findings relate specifically to lupus-associated endocarditis and should not be extrapolated directly to cancer-associated NBTE. Three-dimensional TEE may improve detection and characterization of vegetations compared with two-dimensional TEE, as demonstrated in another study of Libman-Sacks endocarditis [12]. Further studies are required to establish the optimal imaging strategy for suspected malignancy-associated NBTE.

Management

Management of NBTE centers on treatment of the underlying disease and systemic anticoagulation. The 2023 ESC guideline emphasizes treating the underlying malignancy or autoimmune condition to help prevent recurrent NBTE. Anticoagulation should be considered in all patients, with treatment individualized according to bleeding risk. Options include low-molecular-weight heparin, unfractionated heparin, and vitamin K antagonists [9].

Evidence supporting direct oral anticoagulants in NBTE remains limited. Although the 2023 ESC guideline reported no supporting data at that time [9], a subsequent retrospective cohort found no statistically significant differences in thromboembolic events or mortality between anticoagulant classes. These observational findings remain hypothesis-generating and require prospective confirmation [13].

The role of surgery remains uncertain. The ESC guideline advises considering surgery in patients with severe valve dysfunction or large vegetations, with decisions informed by the underlying disease, prognosis, and operative risk [9].

In this patient, anticoagulation was particularly challenging because of the competing risks of recurrent thrombosis and intracranial hemorrhage following mechanical thrombectomy. Despite treatment with different antithrombotic agents, recurrent thromboembolic events continued to occur, highlighting the severity of the underlying malignancy-associated prothrombotic state.

The diagnosis of metastatic pancreatic adenocarcinoma also fundamentally altered the therapeutic context. Given the extent of metastatic disease and clinical deterioration, definitive oncological treatment was not considered feasible.

## Conclusions

Recurrent thrombosis involving multiple vascular territories should prompt consideration of occult malignancy, particularly when events occur despite appropriate antithrombotic therapy. Recurrent embolic stroke in association with venous thromboembolism should raise suspicion for NBTE, particularly when conventional cardioembolic sources are not identified.

Valvular vegetations create a major diagnostic dilemma in distinguishing IE from NBTE. Echocardiographic appearances alone are insufficient, and diagnosis requires integration of imaging, microbiological, clinical, and oncological findings.

Cancer-associated thrombosis may remain difficult to control despite anticoagulation, particularly in advanced mucin-producing adenocarcinoma and when bleeding limits treatment intensity. Treatment of the underlying malignancy is central to the management of NBTE; when disease-directed treatment is not feasible, management may ultimately focus on palliation and goals of care.
